# Clinical option of pemetrexed-based versus paclitaxel-based first-line chemotherapeutic regimens in combination with bevacizumab for advanced non-squamous non-small-cell lung cancer and optimal maintenance therapy: evidence from a meta-analysis of randomized control trials

**DOI:** 10.1186/s12885-021-08136-5

**Published:** 2021-04-17

**Authors:** Le-Tian Huang, Rui Cao, Yan-Ru Wang, Li Sun, Xiang-Yan Zhang, Yi-Jia Guo, Jian-Zhu Zhao, Shu-Ling Zhang, Wei Jing, Jun Song, Cheng-Bo Han, Jietao Ma

**Affiliations:** Department of Oncology, Shengjing Hospital of China Medical University, Shenyang, 110022 China

**Keywords:** Bevacizumab, First-line treatment, Maintenance treatment, Meta-analysis, Non-small-cell lung cancer, Paclitaxel, Pemetrexed

## Abstract

**Background:**

In the era of immunotherapy, it is still unclear which is the best first-line therapy for patients with oncogenic driver negative advanced non-squamous non-small cell lung cancer (NS-NSCLC) who cannot tolerate immunotherapy, or subsequent therapy for patients with oncogenic driver positive NS-NSCLC whose disease progressed on prior targeted therapy. To assess the optimal choice of first-line and maintenance treatment regimens, we performed a meta-analysis of prospective randomized controlled clinical trials (RCTs) of patients with NS-NSCLC on bevacizumab combined with chemotherapy.

**Methods:**

All eligible RCTs comparing pemetrexed-platinum with or without bevacizumab (PP ± B) and paclitaxel-carboplatin with bevacizumab (PC + B) as a first-line therapy, or comparing bevacizumab plus pemetrexed (Pem + B) and bevacizumab alone (B) as a maintenance treatment for advanced NS-NSCLC, were included after systematically searching web databases and meeting abstracts. The main research endpoints were comparisons of overall survival (OS) and progression-free survival (PFS). The other endpoints were objective response rate (ORR), 1-year PFS rate (PFSR1y) and major grade 3/4 treatment-related adverse events.

**Results:**

Data of 3139 patients from six RCTs were incorporated into analyses. Three RCTs were included in an analysis that compared PP ± B and PC + B as a first-line therapy for advanced NS-NSCLC. Patients treated with first-line PP ± B showed similar OS and ORR, but significantly improved PFS (hazard ratio [HR], 0.88) and PFSR1y (risk ratio [RR], 0.83), as compared to patients treated with PC + B (all *P* < 0.05). PP ± B resulted in higher rates of grade 3/4 anemia and thrombocytopenia, but lower rates of neutropenia, febrile neutropenia, and sensory neuropathy than PC + B (all *P* < 0.001). The other three RCTs were included in an analysis that compared Pem + B and B as a maintenance treatment. Compared with B, Pem + B maintenance treatment resulted in significant improvements in OS (HR, 0.88), PFS (HR, 0.64), and PFSR1y (RR, 0.70), but higher rates of anemia, thrombocytopenia, and neutropenia (all *P* < 0.001).

**Conclusion:**

Although the first-line PP + B regimen had longer PFS and PFSR1y than the PC + B regimen, no OS difference was observed. Addition of pemetrexed to bevacizumab as maintenance therapy significantly improved OS compared with bevacizumab maintenance alone, but led to more toxicity.

**Supplementary Information:**

The online version contains supplementary material available at 10.1186/s12885-021-08136-5.

## Background

Lung cancer remains the cancer with the highest incidence and fatality rates worldwide [1]. With the development and clinical application of molecular targeted drugs and immune checkpoint inhibitors, the survival of patients with advanced non-small cell lung cancer (NSCLC) has significantly improved [2, 3]. For patients with oncogenic driver negative non-squamous NSCLC (NS-NSCLC), regimens containing immunotherapy have become a new standard of first-line treatment [4]. Nevertheless, for patients with oncogenic driver (e.g., EGFR, ALK, and ROS1) positive NS-NSCLC whose disease progressed on prior targeted therapy, or patients with oncogenic driver negative NS-NSCLC who cannot tolerate immunotherapy, platinum-based chemotherapy with or without bevacizumab (a monoclonal antibody against vascular endothelial growth factor [VEGF]) remains the recommended first-line or subsequent therapy. Compared with chemotherapy alone, bevacizumab combined with chemotherapy can further prolong progression-free survival (PFS) and overall survival (OS) for patients with NS-NSCLC [5–8]. However, clinicians are still debating the better choice of first-line chemotherapy regimens (pemetrexed+platinum [PP] versus paclitaxel+carboplatin [PC]) in combination with bevacizumab.

In addition, in classic studies of AVAPERL and PARAMOUNT, advanced NS-NSCLC patients with disease control after 4 to 6 cycles of first-line induction chemotherapy can benefit from continuation maintenance treatment with bevacizumab (B), pemetrexed (Pem) or bevacizumab in combination with pemetrexed (Pem + B). However, PFS benefits with doublet maintenance did not translate into an OS advantage [9, 10]. Since two recent trials (COMPASS and EA5508) presented results on single-agent or doublet maintenance therapy at the 2019 American Society of Clinical Oncology meeting [11, 12], we conducted a meta-analysis of randomized control trials (RCTs) to assess the optimal first-line and maintenance regimens for NS-NSCLC patients who are assumed to be intolerant to immunotherapy, by comparing the efficacy and toxicity of first-line treatment regimens between PP ± B and PC + B, and maintenance treatment regimens between Pem + B, Pem, and B.

## Methods

### Search strategy

We identified eligible trials by an electronic search of the Cochrane library, PubMed, Embase, and Web of Science databases using the following terms: “non-small cell lung cancer,” “NSCLC,” and “pemetrexed,” “bevacizumab,” “paclitaxel,” and “chemotherapy,” “clinical trials,” and “maintenance treatment.” The search was performed on March 30, 2020. Two independent reviewers screened titles/abstracts and full text articles. The reference lists including related trials and review articles were manually retrieved.

### Selection criteria

The inclusion criteria were as follows: (1) RCTs; (2) Studies that recruited untreated advanced NS-NSCLC patients; (3) Studies that compared cisplatin (or carboplatin) plus pemetrexed with or without bevacizumab (PP ± B) to carboplatin plus paclitaxel with bevacizumab (PC + B) as first-line treatment, or compared the combination of pemetrexed and bevacizumab to pemetrexed or bevacizumab monotherapy as maintenance treatment. (4) Studies included at least one of the followings as main outcome: OS, PFS, objective response rate (ORR), or grade ≥ 3 treatment-related adverse events (TRAEs). Studies were excluded if they were repeated published studies, non-RCT studies, or non-first-line therapy studies.

### Data collection and quality assessment

Characteristics of trials extracted were: first author’s name, year of publication, patient characteristics, study name, study design and phase, sample size, treatment regimens of the study and control groups, maintenance regimens, and treatment cycles. Endpoints extracted were median PFS (mPFS), median OS (mOS), ORR, and grade ≥ 3 TRAEs. Engauge Digitizer 10.8 software (produced by Mark Mitchell 2014; https://github.com/markummitchell/engauge-digitizer) was used to extract hazard ratio (HR) and 95% confidence intervals (CI), as well as other details (such as numbers at risk) from survival curves if no detailed HR values or numbers at risk were given. Trial quality was assessed with the methods recommended by the Cochrane Collaboration for assessing risk of bias [13]. The criteria used for quality assessment were randomization sequence generation, allocation concealment, blinding of participants and personnel, blinding of outcome assessment, incomplete outcome data, selective reporting, and other biases. Each item was categorized as having high, low, or unclear risk. Sensitivity analysis was performed for the primary outcome with the leave-one-out approach.

### Statistical analysis

The meta-analysis was performed using STATA 12.0 (StataCorp, College Station). Analyses were stratified by trial. We compared the efficacy of each treatment regimen during the induction and maintenance phases. The evaluation included OS, PFS, ORR, and TRAEs. OS was evaluated from the beginning of randomized therapy until death due to any cause. PFS was defined as the beginning of randomized therapy until first event (progression or death from any cause). PFS and OS were expressed as HRs. The ORR, PFSR1y, and the rate of grade ≥ 3 TRAEs were expressed as risk ratios (RRs). All *p*-values were two-sided and were considered statistically significant at the 0.05 level. Heterogeneity was assessed with χ2 test (α = 0.1) and I^2^ statistics. When statistics heterogeneity did not exist among studies (*P* > 0.10, I^2^ < 50%), we used a fixed-effect model; if heterogeneity did exist (*P* < 0.10, I^2^ > 50%), we found the cause and changed to a random-effect model.

## Results

### Characteristics of included trials

**Fig. 1:**
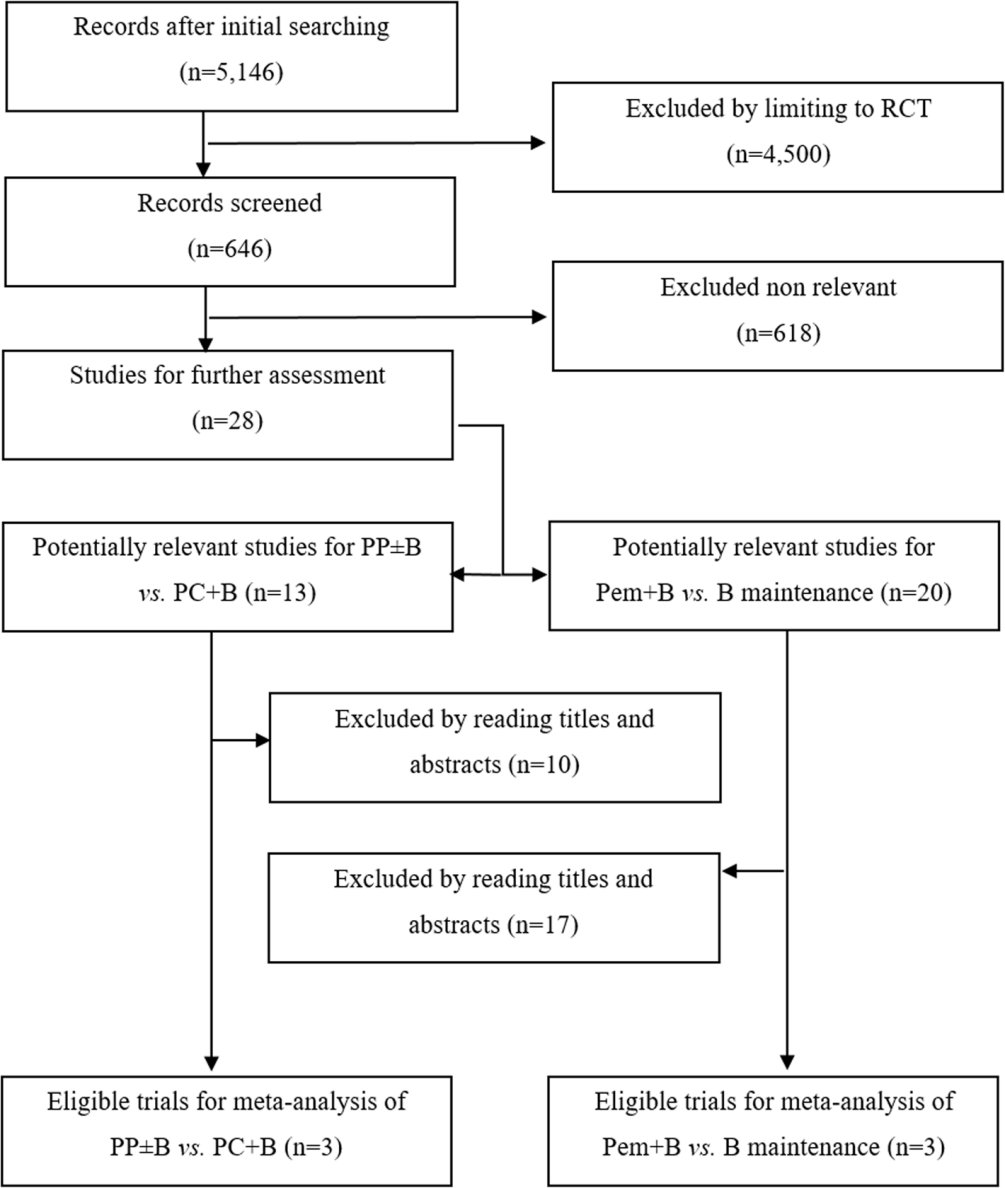
Overview of study search and selection. A list of excluded papers after reading titles and abstracts can be found in additional file 1

Based on the inclusion and exclusion criteria, six RCTs [9, 11, 12, 14–16], including 3144 NS-NSCLC patients were included in this meta-analysis. The baseline characteristics of the included studies are in Tables 1 and 2. Among them, three trials [14–16] were included in analysis comparing first-line treatment regimens between PP ± B and PC + B. Three other trials [9, 11, 12] were included for analysis to compare maintenance regimens between Pem + B and B. The flow diagram of the literature retrieval and selection is in Fig. 1.

**Table 1:** Characteristics of the included studies

Author/published year	Induction arms (randomized)	Induction sample size	Induction regimen	Induction Cycles	Dose of Bevacizumab	Maintenance arms (randomized)	Maintenance sample size	Primary endpoint	Secondary endpoint
Patel, 2013 (PointBreak) [14]	Pem + Cb + Bev (randomized)	442	Pem 500 mg/m2, Cb AUC 6, Bev 15 mg/kg, day 1 every 3 weeks	4	15 mg/kg	Pem + Bev	292	OS	PFS, ORR, DCR, TTPD, toxicity
	Pac + Cb + Bev (randomized)	443	Pac 200 mg/m2, Cb AUC 6, Bev 15 mg/kg, day 1 every 3 weeks			Bev	298		–
Zinner, 2015 (PRONOUNCE) [15]	Pem + Cb (randomized)	182	Pem 500 mg/m2, Cb AUC 6, day 1 every 3 weeks	4	15 mg/kg	Pem	121	PFS without grade 4 toxicity	PFS, OS, ORR, DCR, safety
	Pac + Cb + Bev (randomized)	179	Pac 200 mg/m2, Cb AUC 6, Bev 15 mg/kg, day 1 every 3 weeks			Bev	113		
Galetta, 2015 (ERACLE) [16]	Pem + Cis (randomized)	60	Pem 500 mg/m2, Cis 75 mg/m2, day 1 every 3 weeks	6	15 mg/kg	Pem	44	QOL	PFS, OS, ORR, safety
	Pac + Cb + Bev (randomized)	58	Pac 200 mg/m2, Cb AUC 6 Bev 15 mg/kg, day 1 every 3 weeks			Bev	30		
Barlesi, 2014 (AVAPERL)	Pem + Cis + Bev	376	Pem 500 mg/m2, Cis 75 mg/m2, Bev 7.5 mg/kg, day 1 every 3 weeks	4	7.5 mg/kg	Pem + Bev (randomized)	128	PFS	OS, ORR, DOR
						Bev (randomized)	125		
Ramalingam, 2019 (EA5508) [12]	Pac + Cb + Bev	1432	Pac 200 mg/m2, Cb AUC 6, Bev 15 mg/kg, day 1 every 3 weeks	4	15 mg/kg	Bev (randomized)	287	OS	PFS, ORR, safety
						Pem (randomized)	294		
						Pem + Bev (randomized)	293		
Seto, 2019 (COMPASS) [11]	Pem + Cb + Bev	907	Pem 500 mg/m2, Cb AUC 6, Bev 15 mg/kg, day 1 every 3 weeks	4	15 mg/kg	Bev (randomized)	295	OS (from Random Assignment)	PFS and OS (from induction therapy), safety
						Pem + Bev (randomized)	299		

Abbreviation: *AUC* Area under the curve; *Bev* Bevacizumab; *Cb* Carboplatin; *Cis* Cisplatin; *DCR* Disease control rate; *DOR* Duration of response; *ORR* Objective response rate; *OS* Overall survival; *Pac* Paclitaxel; *Pem* Pemetrexed; *PFS* Progress free survival; *QOL* Quality of life; *TTPD* Time to progressive disease

**Table 2:** Clinicopathological characteristics of the included patients

Study	Randomized arms	Age, Median (y)	Stage IV (%)	Male (%)	ECOG PS 1 (%)	Never Smoker (%)	Adenocarcinoma (%)
PointBreak	PP + B	64.6	89.8%	53.2%	56.1%	10.6%	80.1%
	PC + B	64.9	90.1%	53.3%	55.6%	12.5%	78.3%
PRONOUNCE	PP	65.8	99.5%	57.7%	52.7%	19.9%	83.5%
	PC + B	65.4	100.0%	58.1%	53.1%	3.9%	76.5%
ERACLE	PP	60.0	95.0%	70.0%	22.0%	22.0%	97.0%
	PC + B	62.0	93.0%	78.0%	21.0%	28.0%	97.0%
AVAPERL	Pem + B	60.0	94.4%	57.6%	46.0%	24.8%	85.6%
	B	60.0	89.2%	56.7%	55.6%	26.1%	91.7%
EA5508	B	65.0	93.0%	49.0%	57.0%	10.0%	91.0%
	Pem	63.0	93.0%	49.0%	54.0%	11.0%	88.0%
	Pem + B	64.0	93.0%	49.0%	55.0%	11.0%	91.0%
COMPASS	Pem + B	65.0	92.2%	73.9%	38.5%	24.7%	96.7%
	B	65.0	90.4%	70.8%	42.0%	20.0%	96.3%

Abbreviation: *PP ± B* Pemetrexed-platinum with or without bevacizumab; *PC + B* Paclitaxel-carboplatin with bevacizumab; *Pem + B* Pemetrexed and bevacizumab; *B* Bevacizumab; *ECOG PS* Eastern Cooperative Oncology Group Performance Status

### Comparisons of first-line therapy between PP ± B and PC + B

Three RCTs including 1418 patients were used to compare the efficacy and safety of PP ± B and PC + B [14–16], in which PP + B and PP subgroups were compared with PC + B. Indirect comparisons between subgroups of PP + B and PP were also analyzed.

#### Efficacy

**Fig. 2:**
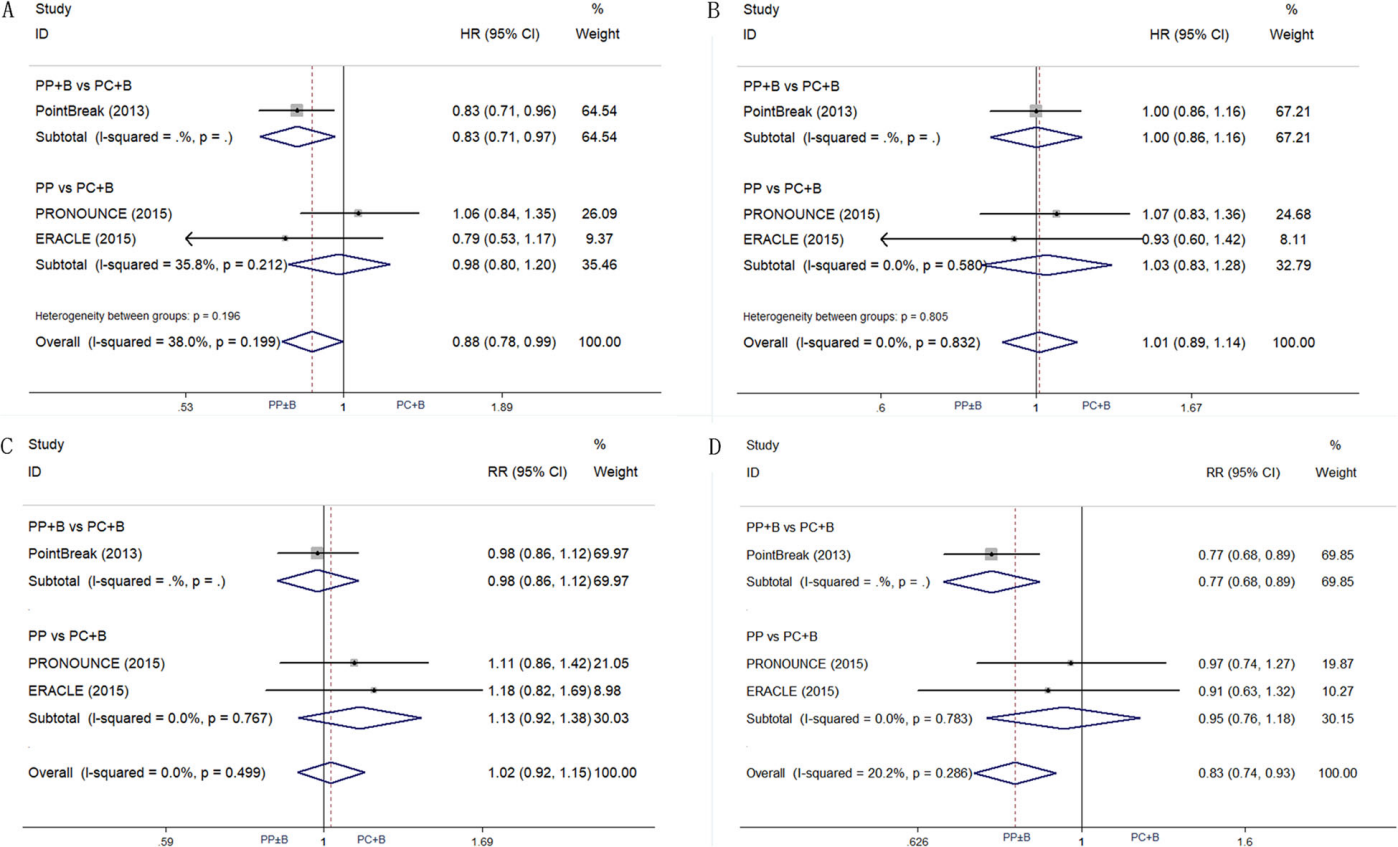
Efficacy comparison of first-line therapy between PP ± B and PC + B. **a**. mPFS; **b**. mOS; **c**. ORR; **d**. PFSR1y. Abbreviations: PP ± B, pemetrexed-platinum with or without bevacizumab; PC + B, paclitaxel-carboplatin with bevacizumab; mPFS, median progression-free survival; mOS, median overall survival; ORR, objective response rates; PFSR1y, 1-year PFS rate

The results of efficacy comparison are in Fig. 2. Compared with PC + B, PP ± B showed a significant benefit in mPFS (HR 0.88; 95% CI, 0.78 to 0.99; *P* = 0.04) and PFSR1y (RR 0.83; 95% CI, 0.74 to 0.93; *P* = 0.001), no significant differences were seen in mOS (HR 1.01; 95% CI, 0.89 to 1.14; *P* = 0.863), and ORR (RR 1.02; 95% CI, 0.92 to 1.15; *P* = 0.675) between the two groups. We also calculated pooled mPFS and mOS using a weighted average of single study medians because of insufficient data on 95% CI values [17]. For subgroups of PP ± B vs. PC + B, mPFS was 5.77 vs. 5.80 months and mOS was 12.16 vs. 13.04 months.

In the subgroup analysis, compared with PC + B group, a PP + B group showed improved mPFS (HR 0.83; 95% CI, 0.71 to 0.97) and PFSR1y (RR 0.77; 95% CI, 0.68 to 0.89) (all *P* < 0.05), but no significant difference in ORR and mOS was observed between the two groups. A PP subgroup showed no advantage compared with a PC + B group for any parameter. Indirect comparisons found no significant differences between PP + B and PP in mPFS (*P* = 0.36), PFSR1y (*P* = 0.11), mOS (*P* = 0.83), or ORR (*P* = 0.41).

#### Safety

The most common grade ≥ 3 TRAEs were hematologic toxicities, hypertension, and sensory neuropathy. Compared with PC + B, PP ± B had a significantly higher risk of anemia (RR 1.75; 95% CI, 1.58 to 1.95; *P* < 0.001) and thrombocytopenia (RR 1.70; 95% CI, 1.47 to 1.96; *P* < 0.001), but a significantly lower risk of neutropenia (RR 0.67; 95% CI, 0.59 to 0.77; *P* = 0.000), febrile neutropenia (RR 0.47; 95% CI, 0.25 to 0.90; *P* = 0.023), and sensory neuropathy (RR 0.21; 95% CI, 0.06 to 0.76; *P* = 0.017). No significant differences were seen in hypertension (*P* = 0.117) or drug-related death (*P* = 0.491) between the two groups (Table 3).

**Table 3:** Summary of forest plot for TRAEs (PP ± B vs. PC + B)

TRAEs	PP ± B n/N (%)	PC + B n/N (%)	Heterogeneity I^2^	Heterogeneity *P* value	RR (95% CI)	*P* value
Drug–related deaths	9/684 (1.3%)	13/680 (1.9%)	0%	0.845	0.84 (0.52,1.37)	0.491
Grade 3/4 TRAEs						
Anemia	97/684 (14.2%)	23/680 (3.4%)	2.3%	0.359	1.75 (1.58,1.95)	0.000
Hypertension	15/684 (2.2%)	29/680 (4.3%)	0%	0.546	0.73 (0.49,1.08)	0.117
Neutropenia	161/684 (23.5%)	267/680 (39.3%)	1.0%	0.364	0.67 (0.59,0.77)	0.000
Thrombocytopenia	144/684 (21.1%)	42/680 (6.2%)	25.3%	0.262	1.70 (1.47,1.96)	0.000
Sensory neuropathy	1/684 (0.1%)	26/680 (3.8%)	0%	0.384	0.21 (0.06,0.76)	0.017
Febrile neutropenia	6/684 (0.9%)	22/680 (3.2%)	0%	0.875	0.47 (0.25,0.90)	0.023

Abbreviation: *PP ± B* Pemetrexed-platinum with or without bevacizumab; *PC + B* paclitaxel-carboplatin with bevacizumab; *RR* Risk ratio; *TRAEs* Treatment-related adverse events

### Comparisons of maintenance treatment between Pem + B, Pem and B

Three RCTs including 1726 patients were used to compare the efficacy and safety of Pem + B and B maintenance [9, 11, 12]. Two RCTs used a continuation maintenance regimen in the study design [9, 11], and one study used continuation and switch maintenance regimens [12]. Indirect comparisons between Pem + B versus Pem maintenance and between Pem versus B maintenance were also analyzed.

#### Efficacy

**Fig. 3:**
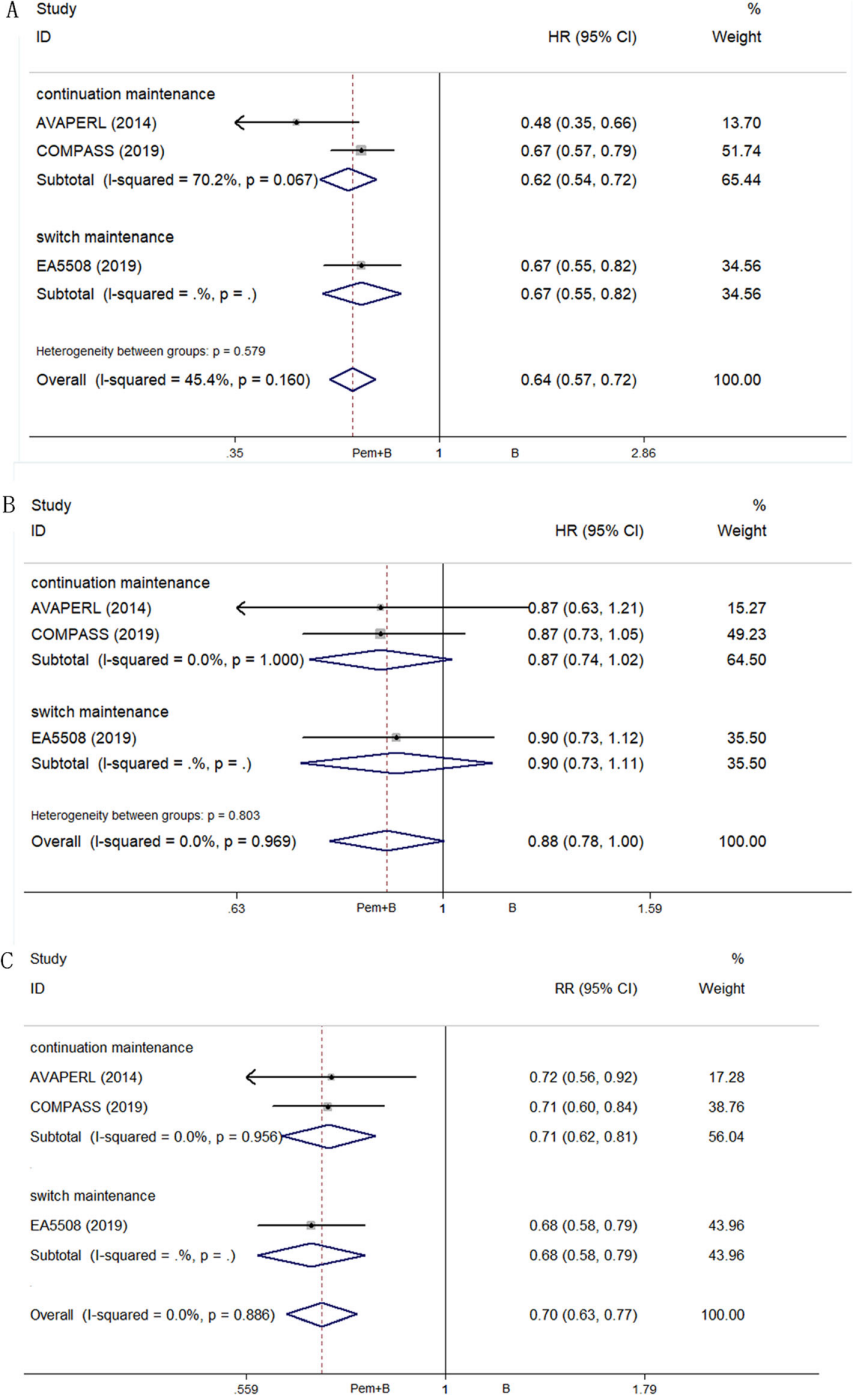
Efficacy comparison of maintenance therapy between Pem + B and B. **a**. mPFS; **b**. mOS; **c**. ORR. Abbreviations: Pem + B, pemetrexed and bevacizumab; B, bevacizumab; mPFS, median progression-free survival; mOS, median overall survival; ORR, objective response rates

The results of efficacy comparison are in Fig. 3. Compared with B alone maintenance, Pem + B maintenance showed significant benefit in mPFS (HR 0.64; 95% CI, 0.57 to 0.72; *P* < 0.001), PFSR1y (RR 0.70; 95% CI, 0.63 to 0.77; *P* < 0.001), and mOS (HR 0.88; 95% CI, 0.78 to 1.00; *P* = 0.05). The mPFS and mOS (calculated using a weighted average of the single study medians) in subgroups Pem + B vs. B were 6.73 vs. 4.03 months and 19.39 vs. 16.36 months, respectively [17]. In subgroup analysis, compared with B maintenance, neither Pem + B continuation maintenance nor Pem + B switch maintenance showed obvious differences in mOS.

Indirect comparisons showed that mPFS (*P* = 0.024) and PFSR1y (odds ratio [OR] 0.57; 95% CI, 0.34 to 0.95; *P* = 0.03) were significantly improved in a Pem + B maintenance group compared with a Pem maintenance group, but with no significant difference in mOS between the two groups (*P* = 0.855). Pem maintenance showed no benefit compared with B maintenance through indirect comparison of PFSR1y (OR 1.22; 95% CI, 0.76 to 1.95; *P* = 0.41).

#### Safety

The most common grade ≥ 3 TRAEs were hematologic toxicities and hypertension. The risk of anemia (RR 1.75; 95% CI, 1.46 to 2.09; *P* < 0.001), neutropenia (RR 1.95; 95% CI, 1.80 to 2.12; P < 0.001), or thrombocytopenia (RR 1.88; 95% CI, 1.55 to 2.28; P < 0.001) were significantly higher in a Pem + B maintenance group than in a B alone maintenance group. No significant difference was observed in hypertension (*P* = 0.864) between the two groups (Table 4).

**Table 4:** Summary of forest plot for grade 3/4 TRAEs (Pem + B vs. B maintenance)

Grade 3/4 TRAEs	Pem + B n/N (%)	B n/N (%)	Heterogeneity I^2^	Heterogeneity *P* value	RR (95%CI)	*P* value
Anemia	35/620 (5.6%)	7/707 (1.0%)	41.8%	0.179	1.75 (1.46, 2.09)	0.000
Hypertension	97/620 (15.6%)	98/707 (13.9%)	60.8%	0.078	1.02 (0.78, 1.34)	0.864
Neutropenia	81/620 (13.1%)	6/707 (0.8%)	0%	0.872	1.95 (1.80, 2.12)	0.000
Thrombocytopenia	14/620 (2.3%)	1/707 (1.0%)	10.4%	0.291	1.88 (1.55, 2.28)	0.000

Abbreviation: *Pem + B* Pemetrexed and bevacizumab; *B* Bevacizumab; *RR* Risk ratio; *TRAEs* Treatment-related adverse events

### Quality of included studies and publication bias

The risk of bias assessment of the included RCTs was low and is shown in Table 5; all studies were of high quality. To minimize publication bias, we executed strict inclusion criteria for selected papers and detected publication bias by several methods. No substantial asymmetry was found by visual inspection of the funnel plots. An Egger linear regression test and Begg rank correlation test also found no evidence of publication bias. Sensitivity analyses were conducted on PFS and OS to assess the heterogeneity in the first-line and maintenance phases. No significant heterogeneity in PFS or OS from any study was found.

**Table 5:** Evaluation of risk of bias in the included studies

Items	PointBreak 2013	AVAPERL 2014	ERACLE 2015	PRONOUNCE 2015	COMPASS 2019	EA5508 2019
Randomization sequence generation	low	low	low	low	low	low
Allocation concealment	unclear	unclear	unclear	unclear	unclear	unclear
Blinding of participants and personnel	high	high	unclear	high	unclear	high
Blinding of outcome assessment	high	high	unclear	high	unclear	unclear
Incomplete outcome data	high	low	high	low	unclear	low
Selective reporting	unclear	unclear	low	low	low	low
Other biases	low	unclear	low	low	unclear	low

## Discussion

Chemotherapy combined with immunotherapy has become the current standard care for patients with negative oncogenic drivers regardless of squamous or non-squamous NSCLC or programmed death ligand 1 (PD-L1) expression level [18]. However, chemotherapy plus bevacizumab is still an important first-line treatment option for patients with oncogenic driver negative NS-NSCLC who cannot tolerate immunotherapy, and is also a subsequent treatment for patients with oncogenic driver positive NS-NSCLC whose disease progressed on prior targeted therapy. Our study enhances understanding of the rational option of first-line chemotherapy regimens in combination with bevacizumab and the subsequent optimal maintenance therapy for these advanced NS-NSCLC cases. In view of the inclusion of the latest large-sample RCTs and strict inclusion criteria, this meta-analysis thus answers some controversial questions that have not been solved in previous studies.

One question is which first-line chemotherapy regimen (pemetrexed- versus paclitaxel-based) is a better choice when used in combination with bevacizumab. Bevacizumab combined with platinum-based doublet chemotherapy shows clinical benefits for advanced NS-NSCLC in multiple RCTs, with mPFS of 6.2–9.2 months and mOS of 12.3–24.3 months [5–8]. A meta-analysis showed comparable efficacy for taxane and non-taxane regimens in combination with bevacizumab for treatment of patients with NS-NSCLC. For taxane and non-taxane groups, respective weighted mOS was 14.4 and 13.7 months (*P* = 0.5), mPFS was 6.93 and 6.99 months (*P* = 0.61), and ORR was 41 and 39% (*P* = 0.65) [19]. Our meta-analysis found that PP ± B had a significant benefit for PFS and PFSR1y, but no difference in OS and ORR between PP ± B and PC + B. For subgroup comparisons with PC + B, PP + B had significant benefits for PFS and PFSR1y, but not OS. The negative OS outcome may be attributed to the subsequent maintenance treatment options. Among three studies included for comparison of first-line treatments, PRONOUNCE and ERACLE studies used Pem alone as maintenance therapy; only the PointBreak study used Pem + B maintenance [14–16]. In our meta-analysis, the two groups had different grade 3/4 toxicity profiles. In the PP ± B group, the risk of severe anemia was 1.75 times and the risk of thrombocytopenia was 1.7 times that in the PC + B group. In the PC + B group, the risk of severe sensory neuropathy was 4.76 times and the risk of febrile neutropenia was 2.13 times that in the PP ± B group (Table 3). Since we saw no significant difference in OS between the two groups, the tolerance of patients to different drug toxicities should be considered when choosing first-line chemotherapies. That is, the choice of first-line chemotherapy mainly depends on differences in toxicity profiles.

The second question is which maintenance therapy (B versus Pem + B) is preferred. Maintenance therapy has emerged as a confirmed treatment strategy for advanced NSCLC. For NS-NSCLC patients, Pem + B in combination or as a single drug as a maintenance therapy is shown to be beneficial for survival [9, 10, 14, 20]. Even though Pem + B showed significant benefits in PFS compared to monotherapy B maintenance, four previous studies did not recommend Pem + B as a standard maintenance regimen because of the lack of OS benefits and higher toxicity [9, 11, 12, 14]. Combining two recent RCTs [11, 12], our meta-analysis showed not only an improvement in PFS with Pem + B maintenance, but also a benefit in OS (*P* = 0.05), and the latter was demonstrated for the first time. The PointBreak study showed a longer OS for Pem + B maintenance than B alone, but that trial could not be included in our meta-analysis, because the timepoint after random assignment was different from those in the other trials [14]. Although the addition of pemetrexed to bevacizumab as a maintenance therapy (Pem + B) can moderately improve survival, we still need to be cautious, as doublet maintenance leads to more toxicities, especially hematological toxicity. In our meta-analysis, the risk of grade 3/4 TRAEs including anemia, thrombocytopenia, and neutropenia were all significantly higher in the Pem + B groups. This may lead to a prolonged treatment interval, poor compliance with maintenance treatment, or even drug-related termination or death. Therefore, we recommend that only patients with NS-NSCLC with controlled disease after 4 to 6 cycles of PP + B induction therapy who have not experienced intolerable toxicity receive Pem + B continuation maintenance therapy whenever possible.

The third question is whether bevacizumab should be added to a PP regimen. Pemetrexed combined with platinum is the preferred frontline chemotherapy for patients with NS-NSCLC in National Comprehensive Cancer Network (NCCN) guidelines [21]. Efficacy of PP + B has been observed in some trials [9, 11, 14, 20], but no direct prospective comparison has been made between PP + B and PP. However, designing prospective trials comparing PP + B and PP seems increasingly infeasible. In both the PRONOUNCE and ERACLE study designs, bevacizumab was added to the PC regimen, but not to the PP regimen. Nevertheless, no significant difference in PFS or OS was observed between PP and PC + B [15, 16]. Our meta-analysis indicated that PP + B significantly prolonged PFS, as compared to PC + B, but no significant differences were seen in any survival data between PP + B and PP by indirect comparisons. However, the strength of the evidence to clarify this issue remains limited.

Currently, pembrolizumab in combination with chemotherapy is the preferred first-line regimen according to NCCN guidelines for patients with oncogenic driver negative NS-NSCLC and without contraindications to PD-1/PD-L1 inhibitors, regardless of PD-L1 expression level. Atezolizumab in combination with chemotherapy and bevacizumab is the other recommended regimen [22]. Interestingly, the chemotherapies in these two regimens differ (carboplatin/cisplatin+pemetrexed, and carboplatin+paclitaxel, respectively). In the future, we should focus on whether bevacizumab is a good partner to combine with chemotherapy and anti-PD-1 immunotherapy (e.g., pembrolizumab) for both first-line and maintenance treatment.

In our meta-analysis, we strictly limited the inclusion criteria to RCTs. However, summary statistics rather than individual patient data were used for each trial, and the studies included were heterogeneous, with varying patient populations and different study designs. For example, EGFR-sensitizing mutation populations were excluded in the COMPASS trial, but not mentioned in the other five trials. This difference may lead to different subsequent line regimens and survival.

## Conclusions

On the basis of the answers to these three questions, we have made preliminary recommendations for first-line and maintenance treatment strategies for patients with advanced NS-NSCLC with negative drivers who cannot tolerate immunotherapy: i) PP + B as first-line therapy is as effective as PC + B in patients with advanced NS-NSCLC, and the toxicity profile of the two therapies varies. ii) Addition of pemetrexed to bevacizumab as maintenance therapy significantly improved survival, but led to more toxicity. iii) Patients’ tolerance and toxicity profiles should be considered when choosing treatment regimens. iv) Treatment with PP + B or PC + B followed by Pem + B rather than single-drug B or Pem maintenance might be the best choice under the premise of tolerable toxicity.

In addition, a phase III RCT IMpower-150 found that in a subset of EGFR/ALK-positive advanced NS-NSCLC patients whose disease progressed on prior targeted therapy, adding atezolizumab to PC + B can significantly improve the survival. Although additional studies on quadruple use focused specifically on this subgroup of patients are still needed, at least for now, the above recommendations may also be applicable for patients with positive oncogenic drivers whose disease progressed on prior targeted therapy.

## Supplementary Information

**Additional file 1:.** A list of excluded papers after reading titles and abstracts.

## Data Availability

The data that support the findings of this study are available from the corresponding author upon reasonable request.

## Abbreviations

NSCLC: Non-small cell lung cancer
NS-NSCLC: Non-squamous NSCLC
PD-L1: Programmed death-ligand 1
RCTs: Randomized controlled clinical trials
VEGF: Vascular endothelial growth factor
PP ± B: Pemetrexed-platinum with or without bevacizumab
PC + B: Paclitaxel-carboplatin with bevacizumab
Pem + B: Pemetrexed and bevacizumab
TRAEs: Treatment-related adverse events
CI: Confidence intervals
ORR: Objective response rate
OS: Overall survival
PFS: Progress free survival
PFSR1y: 1-Year PFS rate
HR: Hazard ratio
RR: Risk ratio
NCCN: National comprehensive cancer network
