# Supplementary material for: Clinical option of pemetrexed-based versus paclitaxel-based first-line chemotherapeutic regimens in combination with bevacizumab for advanced non-squamous non-small-cell lung cancer and optimal maintenance therapy: evidence from a meta-analysis of randomized control trials
Source: BMC Cancer. 2021 Apr 17;21:426. doi: 10.1186/s12885-021-08136-5 (PMC8052669; doi:10.1186/s12885-021-08136-5)
Supplement: Supplementary file 1 — Additional file 1:. A list of excluded papers after reading titles and abstracts. [file 12885_2021_8136_MOESM1_ESM.docx]

**A list of excluded papers after reading titles and abstracts (n=10).**

1: Ramalingam SS, Dahlberg SE, Belani CP, Saltzman JN, Pennell NA, Nambudiri GS, McCann JC, Winegarden JD, Kassem MA, Mohamed MK, Rothman JM, Lyss AP, Horn L, Stinchcombe TE, Schiller JH. Pemetrexed, Bevacizumab, or the Combination As Maintenance Therapy for Advanced Nonsquamous Non-Small-Cell Lung Cancer: ECOG-ACRIN 5508. J Clin Oncol. 2019 Sep 10;37(26):2360-2367. doi: 10.1200/JCO.19.01006. Epub 2019 Jul 30. PMID: 31361535; PMCID: PMC7001786.

2: Wakelee HA, Dahlberg SE, Keller SM, Tester WJ, Gandara DR, Graziano SL, Adjei AA, Leighl NB, Aisner SC, Rothman JM, Patel JD, Sborov MD, McDermott SR, Perez-Soler R, Traynor AM, Butts C, Evans T, Shafqat A, Chapman AE, Kasbari SS, Horn L, Ramalingam SS, Schiller JH; ECOG-ACRIN. Adjuvant chemotherapy with or without bevacizumab in patients with resected non-small-cell lung cancer (E1505): an open-label, multicentre, randomised, phase 3 trial. Lancet Oncol. 2017 Dec;18(12):1610-1623. doi: 10.1016/S1470-2045(17)30691-5. Epub 2017 Nov 9. PMID: 29129443; PMCID: PMC5789803.

3: Wakelee H, Zvirbule Z, De Braud F, Kingsley CD, Mekhail T, Lowe T, Schütte W,

Lena H, Lawler W, Braiteh F, Cosgriff T, Kaen D, Boyer M, Hsu J, Phan S, Novello

S. Efficacy and Safety of Onartuzumab in Combination With First-Line Bevacizumab- or Pemetrexed-Based Chemotherapy Regimens in Advanced Non-Squamous Non-Small-Cell Lung Cancer. Clin Lung Cancer. 2017 Jan;18(1):50-59. doi: 10.1016/j.cllc.2016.09.013. Epub 2016 Oct 19. PMID: 27856142.

4: Spigel DR, Patel JD, Reynolds CH, Garon EB, Hermann RC, Govindan R, Olsen MR, Winfree KB, Chen J, Liu J, Guba SC, Socinski MA, Bonomi P. Quality of life

analyses from the randomized, open-label, phase III PointBreak study of

pemetrexed-carboplatin-bevacizumab followed by maintenance pemetrexed-

bevacizumab versus paclitaxel-carboplatin-bevacizumab followed by maintenance

bevacizumab in patients with stage IIIB or IV nonsquamous non-small-cell lung

cancer. J Thorac Oncol. 2015 Feb;10(2):353-9. doi: 10.1097/JTO.0000000000000277.

PMID: 25611228.

5: Patel JD, Bonomi P, Socinski MA, Govindan R, Hong S, Obasaju C, Pennella EJ,

Girvan AC, Guba SC. Treatment rationale and study design for the pointbreak

study: a randomized, open-label phase III study of pemetrexed/carboplatin/bevacizumab followed by maintenance

pemetrexed/bevacizumab versus paclitaxel/carboplatin/bevacizumab followed by

maintenance bevacizumab in patients with stage IIIB or IV nonsquamous non-small-

cell lung cancer. Clin Lung Cancer. 2009 Jul;10(4):252-6. doi:

10.3816/CLC.2009.n.035. PMID: 19632943.

6: Zinner RG, Saxman SB, Peng G, Monberg MJ, Ortuzar WI. Treatment rationale

and study design for a randomized trial of pemetrexed/carboplatin followed by

maintenance pemetrexed versus paclitaxel/carboplatin/bevacizumab followed by

maintenance bevacizumab in patients with advanced non-small-cell lung cancer of

nonsquamous histology. Clin Lung Cancer. 2010 Sep 1;11(5):352-7. doi:

10.3816/CLC.2010.n.045. PMID: 20837462.

7: Galetta D, Pisconti S, Cinieri S, Pappagallo GL, Gebbia V, Borsellino N,

Maiello E, Rinaldi A, Montrone M, Rizzo P, Marzano N, Sasso N, Febbraro A,

Colucci G. Induction pemetrexed and cisplatin followed by maintenance pemetrexed

versus carboplatin plus paclitaxel plus bevacizumab followed by maintenance

bevacizumab: a quality of life-oriented randomized phase III study in patients

with advanced non-squamous non-small-cell lung cancer (ERACLE). Clin Lung

Cancer. 2011 Nov;12(6):402-6. doi: 10.1016/j.cllc.2011.06.006. Epub 2011 Aug 10.

PMID: 21831718.

8: Reynolds CH, Patel JD, Garon EB, Olsen MR, Bonomi P, Govindan R, Pennella

EJ, Liu J, Guba SC, Li S, Spigel DR, Hermann RC, Socinski MA, Obasaju CK.

Exploratory Subset Analysis of African Americans From the PointBreak Study:

Pemetrexed-Carboplatin-Bevacizumab Followed by Maintenance Pemetrexed-

Bevacizumab Versus Paclitaxel-Carboplatin-Bevacizumab Followed by Maintenance

Bevacizumab in Patients With Stage IIIB/IV Nonsquamous Non-Small-Cell Lung

Cancer. Clin Lung Cancer. 2015 May;16(3):200-8. doi: 10.1016/j.cllc.2014.11.004.

Epub 2014 Nov 18. PMID: 25516338.

9: Gridelli C, Bennouna J, de Castro J, Dingemans AM, Griesinger F, Grossi F,

Rossi A, Thatcher N, Wong EK, Langer C. Randomized phase IIIb trial evaluating

the continuation of bevacizumab beyond disease progression in patients with advanced non-squamous non-small-cell lung cancer after first-line treatment with bevacizumab plus platinum-based chemotherapy: treatment rationale and protocol dynamics of the AvaALL (MO22097) trial. Clin Lung Cancer. 2011 Nov;12(6):407-11. doi: 10.1016/j.cllc.2011.05.002. Epub 2011 Jun 25. PMID: 21705281.

10: Klein R, Muehlenbein C, Liepa AM, Babineaux S, Wielage R, Schwartzberg L.

Cost-effectiveness of pemetrexed plus cisplatin as first-line therapy for advanced nonsquamous non-small cell lung cancer. J Thorac Oncol. 2009 Nov;4(11):1404-14. doi: 10.1097/JTO.0b013e3181ba31e0. PMID: 19786904.

**A list of excluded papers after reading titles and abstracts (n=17).**

1: Patel JD, Socinski MA, Garon EB, Reynolds CH, Spigel DR, Olsen MR, Hermann

RC, Jotte RM, Beck T, Richards DA, Guba SC, Liu J, Frimodt-Moller B, John WJ,

Obasaju CK, Pennella EJ, Bonomi P, Govindan R. PointBreak: a randomized phase

III study of pemetrexed plus carboplatin and bevacizumab followed by maintenance

pemetrexed and bevacizumab versus paclitaxel plus carboplatin and bevacizumab

followed by maintenance bevacizumab in patients with stage IIIB or IV nonsquamous non-small-cell lung cancer. J Clin Oncol. 2013 Dec 1;31(34):4349-57.

doi: 10.1200/JCO.2012.47.9626. Epub 2013 Oct 21. PMID: 24145346; PMCID:

PMC4881367.

2: Barlesi F, Scherpereel A, Rittmeyer A, Pazzola A, Ferrer Tur N, Kim JH, Ahn

MJ, Aerts JG, Gorbunova V, Vikström A, Wong EK, Perez-Moreno P, Mitchell L,

Groen HJ. Randomized phase III trial of maintenance bevacizumab with or without

pemetrexed after first-line induction with bevacizumab, cisplatin, and pemetrexed in advanced nonsquamous non-small-cell lung cancer: AVAPERL (MO22089). J Clin Oncol. 2013 Aug 20;31(24):3004-11. doi:10.1200/JCO.2012.42.3749. Epub 2013 Jul 8. PMID: 23835708.

3: Zinner RG, Obasaju CK, Spigel DR, Weaver RW, Beck JT, Waterhouse DM, Modiano MR, Hrinczenko B, Nikolinakos PG, Liu J, Koustenis AG, Winfree KB, Melemed SA, Guba SC, Ortuzar WI, Desaiah D, Treat JA, Govindan R, Ross HJ. PRONOUNCE: randomized, open-label, phase III study of first-line pemetrexed + carboplatin followed by maintenance pemetrexed versus paclitaxel + carboplatin + bevacizumab followed by maintenance bevacizumab in patients ith advanced nonsquamous non-small-cell lung cancer. J Thorac Oncol. 2015 Jan;10(1):134-42. doi: 10.1097/JTO.0000000000000366. PMID: 25371077; PMCID: PMC4276572.

4: Karayama M, Inui N, Fujisawa T, Enomoto N, Nakamura Y, Kuroishi S, Yokomura

K, Koshimizu N, Sato M, Toyoshima M, Shirai T, Masuda M, Yamada T, Imokawa S,

Suda T. Maintenance therapy with pemetrexed and bevacizumab versus pemetrexed

monotherapy after induction therapy with carboplatin, pemetrexed, and bevacizumab in patients with advanced non-squamous non small cell lung cancer. Eur J Cancer. 2016 May;58:30-7. doi: 10.1016/j.ejca.2016.01.013. Epub 2016 Feb 27. PMID: 26922170.

5: Spigel DR, Patel JD, Reynolds CH, Garon EB, Hermann RC, Govindan R, Olsen

MR, Winfree KB, Chen J, Liu J, Guba SC, Socinski MA, Bonomi P. Quality of life

analyses from the randomized, open-label, phase III PointBreak study of

pemetrexed-carboplatin-bevacizumab followed by maintenance pemetrexed-

bevacizumab versus paclitaxel-carboplatin-bevacizumab followed by maintenance

bevacizumab in patients with stage IIIB or IV nonsquamous non-small-cell lung

cancer. J Thorac Oncol. 2015 Feb;10(2):353-9. doi: 10.1097/JTO.0000000000000277.

PMID: 25611228.

6: Reynolds CH, Patel JD, Garon EB, Olsen MR, Bonomi P, Govindan R, Pennella

EJ, Liu J, Guba SC, Li S, Spigel DR, Hermann RC, Socinski MA, Obasaju CK.

Exploratory Subset Analysis of African Americans From the PointBreak Study:

Pemetrexed-Carboplatin-Bevacizumab Followed by Maintenance Pemetrexed-

Bevacizumab Versus Paclitaxel-Carboplatin-Bevacizumab Followed by Maintenance

Bevacizumab in Patients With Stage IIIB/IV Nonsquamous Non-Small-Cell Lung

Cancer. Clin Lung Cancer. 2015 May;16(3):200-8. doi: 10.1016/j.cllc.2014.11.004.

Epub 2014 Nov 18. PMID: 25516338.

7: Rittmeyer A, Gorbunova V, Vikström A, Scherpereel A, Kim JH, Ahn MJ, Chella

A, Chouaid C, Campbell AK, Barlesi F. Health-related quality of life in patients

with advanced nonsquamous non-small-cell lung cancer receiving bevacizumab or

bevacizumab-plus-pemetrexed maintenance therapy in AVAPERL (MO22089). J Thorac Oncol. 2013 Nov;8(11):1409-16. doi: 10.1097/JTO.0b013e3182a46bcf. PMID: 24077452.

8: Stevenson JP, Langer CJ, Somer RA, Evans TL, Rajagopalan K, Krieger K,

Jacobs-Small M, Dyanick N, Milcarek B, Coakley S, Walker S, Eaby-Sandy B,

Hageboutros A. Phase 2 trial of maintenance bevacizumab alone after bevacizumab

plus pemetrexed and carboplatin in advanced, nonsquamous nonsmall cell lung

cancer. Cancer. 2012 Nov 15;118(22):5580-7. doi: 10.1002/cncr.27576. Epub 2012

Apr 27. PMID: 22544579.

9: Casey EM, Harb W, Bradford D, Bufill J, Nattam S, Patel J, Fisher W, Latz JE,

Li X, Wu J, Hanna N. Randomized, double-blinded, multicenter, phase II study of

pemetrexed, carboplatin, and bevacizumab with enzastaurin or placebo in

chemonaïve patients with stage IIIB/IV non-small cell lung cancer: Hoosier

Oncology Group LUN06-116. J Thorac Oncol. 2010 Nov;5(11):1815-20. doi:

10.1097/JTO.0b013e3181ee820c. PMID: 20881647.

10: Patel JD, Bonomi P, Socinski MA, Govindan R, Hong S, Obasaju C, Pennella EJ,

Girvan AC, Guba SC. Treatment rationale and study design for the pointbreak study: a randomized, open-label phase III study of pemetrexed/carboplatin/bevacizumab followed by maintenance pemetrexed/bevacizumab versus paclitaxel/carboplatin/bevacizumab followed by maintenance bevacizumab in patients with stage IIIB or IV nonsquamous non-small-cell lung cancer. Clin Lung Cancer. 2009 Jul;10(4):252-6. doi:10.3816/CLC.2009.n.035. PMID: 19632943.

11: Casey EM, Harb W, Bradford D, Bufill J, Nattam S, Patel J, Fisher W, Latz

JE, Li X, Wu J, Hanna N. Randomized, double-blinded, multicenter, phase II study

of pemetrexed, carboplatin, and bevacizumab with enzastaurin or placebo in

chemonaïve patients with stage IIIB/IV non-small cell lung cancer: Hoosier

Oncology Group LUN06-116. J Thorac Oncol. 2010 Nov;5(11):1815-20. doi:

10.1097/JTO.0b013e3181ee820c. PMID: 20881647.

12: Dy GK, Molina JR, Qi Y, Ansari R, Thomas S, Ross HJ, Soori G, Anderson D,

Aubry MC, Meyers J, Adjei AA, Mandrekar S, Adjei AA. NCCTG N0821 (Alliance): a phase II first-line study of pemetrexed, carboplatin, and bevacizumab in elderly

patients with advanced nonsquamous non-small-cell lung cancer with good

performance status. J Thorac Oncol. 2014 Aug;9(8):1146-53. doi:

10.1097/JTO.0000000000000217. PMID: 25157767; PMCID: PMC4145612.

13: Stevenson JP, Langer CJ, Somer RA, Evans TL, Rajagopalan K, Krieger K,

Jacobs-Small M, Dyanick N, Milcarek B, Coakley S, Walker S, Eaby-Sandy B,

Hageboutros A. Phase 2 trial of maintenance bevacizumab alone after bevacizumab

plus pemetrexed and carboplatin in advanced, nonsquamous nonsmall cell lung

cancer. Cancer. 2012 Nov 15;118(22):5580-7. doi: 10.1002/cncr.27576. Epub 2012

Apr 27. PMID: 22544579.

14: Galetta D, Pisconti S, Cinieri S, Pappagallo GL, Gebbia V, Borsellino N,

Maiello E, Rinaldi A, Montrone M, Rizzo P, Marzano N, Sasso N, Febbraro A,

Colucci G. Induction pemetrexed and cisplatin followed by maintenance pemetrexed

versus carboplatin plus paclitaxel plus bevacizumab followed by maintenance

bevacizumab: a quality of life-oriented randomized phase III study in patients

with advanced non-squamous non-small-cell lung cancer (ERACLE). Clin Lung

Cancer. 2011 Nov;12(6):402-6. doi: 10.1016/j.cllc.2011.06.006. Epub 2011 Aug 10.

PMID: 21831718.

15: Gridelli C, Bennouna J, de Castro J, Dingemans AM, Griesinger F, Grossi F,

Rossi A, Thatcher N, Wong EK, Langer C. Randomized phase IIIb trial evaluating

the continuation of bevacizumab beyond disease progression in patients with

advanced non-squamous non-small-cell lung cancer after first-line treatment with

bevacizumab plus platinum-based chemotherapy: treatment rationale and protocol

dynamics of the AvaALL (MO22097) trial. Clin Lung Cancer. 2011 Nov;12(6):407-11. doi: 10.1016/j.cllc.2011.05.002. Epub 2011 Jun 25. PMID: 21705281.

16: Zinner RG, Saxman SB, Peng G, Monberg MJ, Ortuzar WI. Treatment rationale and study design for a randomized trial of pemetrexed/carboplatin followed by maintenance pemetrexed versus paclitaxel/carboplatin/bevacizumab followed by

maintenance bevacizumab in patients with advanced non-small-cell lung cancer of

nonsquamous histology. Clin Lung Cancer. 2010 Sep 1;11(5):352-7. doi:

10.3816/CLC.2010.n.045. PMID: 20837462.

17: Patel JD, Bonomi P, Socinski MA, Govindan R, Hong S, Obasaju C, Pennella EJ,

Girvan AC, Guba SC. Treatment rationale and study design for the pointbreak study: a randomized, open-label phase III study of pemetrexed/carboplatin/bevacizumab followed by maintenance pemetrexed/bevacizumab versus paclitaxel/carboplatin/bevacizumab followed by maintenance bevacizumab in patients with stage IIIB or IV nonsquamous non-small-cell lung cancer. Clin Lung Cancer. 2009 Jul;10(4):252-6. doi:10.3816/CLC.2009.n.035. PMID: 19632943.
